# Synthesis mechanism from graphene quantum dots to carbon nanotubes by ion-sputtering assisted chemical vapor deposition

**DOI:** 10.1186/s11671-024-04027-3

**Published:** 2024-05-07

**Authors:** Jun Mok Ha, Seoung Ho Lee, Daehyeon Park, Young Jun Yoon, In Mok Yang, Junhyeok Seo, Yong Seok Hwang, Chan Young Lee, Jae Kwon Suk, Jun Kue Park, Sunmog Yeo

**Affiliations:** 1 Particle Beam Research Division, Korea Multi-Purpose Accelerator Complex (KOMAC), Korea Atomic Energy Research Institute (KAERI), 181 Mirae-Ro, Geonchon-Eup, Gyeongju-Si, Gyeonbuk 38180 Republic of Korea; 2 Department of Material and Equipment Development, Korea Research Institute of Decommissioning (KRID), 1655 Bulguk-Ro, Munmudaewang-Myeon, Gyeongju-Si, Gyeongbuk 38120 Republic of Korea; 3 Department of Nuclear Engineering, College of Engineering, Ulsan National Institute of Science and Technology (UNIST), 50 UNIST-Gil, Ulsan, 44919 Republic of Korea; 4 Department of Electrical and Electronic Engineering, Hannam University, 70 Hannam-Ro, Daedeok-Gu, Daejeon, 34430 Republic of Korea

**Keywords:** Graphene quantum dots, Carbon nanotubes, Controllable formation, Ion-sputtering, Chemical vapor deposition, Platinum nanoparticles

## Abstract

We present the first work of the synthesis mechanism from graphene quantum dots (GQDs) to carbon nanotubes (CNTs) by an ion-sputtering assisted chemical vapor deposition. During the annealing process, a Pt thin film deposited by the ion-sputtering was dewetted and agglomerated to form many nanometer-sized particles, leading to Pt nanoparticles (PtNPs) that can act as catalysts for creating carbon allotropes. The shape of the allotropes can be effectively tailored from GQDs to CNTs by controlling three key parameters such as the dose of catalytic ions (D), amounts of carbon source (S), and thermal energy (T). In our work, it was clearly proved that the growth control from GQDs to CNTs has a comparably proportional relationship with D and S, but has a reverse proportional relationship with T. Furthermore, high-purity GQDs without any other by-products and the CNTs with the cap of PtNPs were generated. Their shapes were appropriately controlled, respectively, based on the established synthesis mechanism.

**Graphical abstract:**

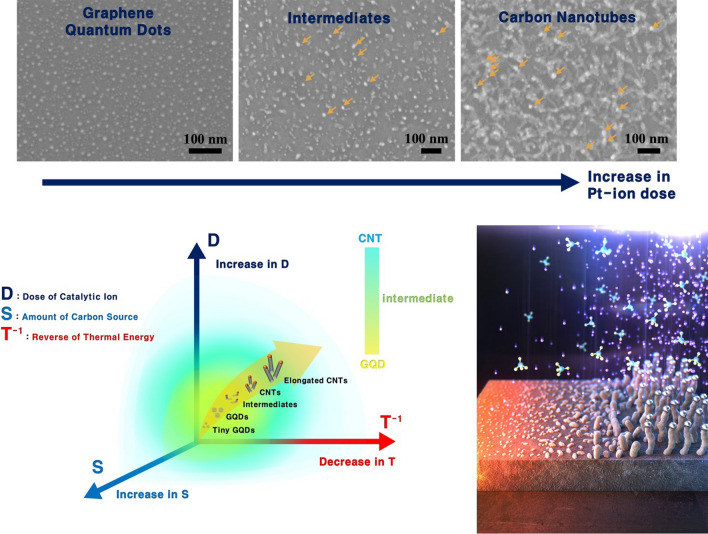

**Supplementary Information:**

The online version contains supplementary material available at 10.1186/s11671-024-04027-3.

## Introduction

Carbon allotropes are one of the most important issues in material science because they have versatile properties and are scientific topics of crucial importance. Among the allotropes, the zero-dimensional graphene quantum dots (GQDs) and the one-dimensional carbon nanotubes (CNTs) have attracted worldwide attention due to their diverse applications [1–9]. For GQDs, their advantages such as stable fluorescence, outstanding biocompatibility, low toxicity, low cost, and high surface area have led to optical displays, sensors, bio-imaging drug delivery, and photo-catalysts [4–6, 10–14]. On the other hand, CNTs have unique mechanical, electrical, and optical properties like high tensile strength, ultra-light weight, special electronic structures, and high chemical and thermal stability. These properties have made them useful for diverse applications such as nano-electronic devices, energy storage devices, composite materials, medicine, air and water filtering, and sensors [7–9, 15–18]. Since GQDs and CNTs are independently important research topics, the synthesis methods for GQDs and CNTs have been developed individually.

The GQDs are generally prepared by top-down and bottom-up methods. The top-down methods represent cutting, exfoliation, electrochemical oxidation, chemical ablation, arc-discharge or plasma treatment, and ultrasonication [4, 12–14, 19–22], while the bottom-up methods include electrochemical carbonization, microwave irradiation synthesis, hydrothermal or solvothermal treatment, and thermal decomposition [23–27]. Although all methods have their unique advantages, they typically use strong oxidants and acids, resulting in their limited application to bio-field and environmental problems [4–6, 12–14, 19–27]. Since most GQDs are fabricated through the solution-based approaches, it is difficult to obtain GQD arrays or patterns for device implementation. However, in our previous work, we introduced a simple and efficient method for creating pure and patterned GQDs using ion-beam assisted chemical vapor deposition (CVD) [28]. On the other hands, unlike the GQD synthesis, CNTs have been mainly produced by dry methods such as arc discharge, laser ablation, and CVD [8, 9, 29–35]. Except for the CVD technology, arc discharge and laser ablation approaches have had difficulties in obtaining CNT arrays or patterns. There have also been some problems with the impurity production and precise control of CNTs [29–32]. Therefore, ion-beam assisted CVD may bridge the individually developed synthesis methods for GQDs and CNTs.

Although many synthesis methods have been developed and studied in depth individually, to the best of our knowledge, no research has been reported that both GQDs and CNTs were successfully synthesized in series by regulating the synthesis parameters. In this paper, we present a simple and convenient route from GQDs to CNTs by an ion-sputtering assisted CVD. Since this method can be easily applied to array and patterning processes, this study is extremely useful when a device requires both properties of GQD and CNT. Furthermore, the opposite electrical characteristics of GQDs and CNTs suggest that by fine-tuning the synthesis parameters, quantum criticality could be achieved at a certain point associated with the metal–insulator transition [36, 37]. This study also offers valuable insights into the topology of transforming zero-dimensional GQDs into one-dimensional CNTs through manipulation of synthesis parameters.

## Experimental section

### Materials

A polished Si (100) wafer and an ethanol (99.999%) solution were purchased from Namkang Hi-tech Co. (Republic of Korea) and Merck Chemicals (Darmstadt, Germany), respectively. A Pt disk (> 99.9%) with a diameter of 50 mm was used as the target of sputtering and purchased from Goodfellow (Huntingdon, UK). Ar (99.999%) and CH_4_ (99.999%) gases were used as the atmospheric and carbon source gases, respectively.

### Synthesis of carbon allotropes

Pt ions were deposited onto a polished Si (100) wafer using a magnetron sputtering system (MSP-30T, Vacuum Device Inc., Japan; Cressington 108auto, Ted Pella, Inc., USA). The energy and dose of Pt ions were 2 keV and 1 × 10^13^–5 × 10^14^/cm^2^, respectively. For the controllable formation of carbon allotropes, a Pt-deposited Si substrate (PtDS) was annealed at different temperatures (700–1100 °C) under a vacuum of 5 × 10^–1^ Torr with Ar gas (100 sccm), which was used as an atmospheric gas. The heating rate was 30 °C/min. During the annealing process, Pt nanoparticles (PtNPs) were fabricated as reported in previous papers [38, 39] and used as catalysts to synthesize carbon allotropes. The temperatures were ramped up to the desired points (700–1100 °C) under an Ar flow at 100 sccm. When the heating temperature reached its highest point, the carbon allotropes were grown on the PtNPs at this temperature for 20 min with a gas mixture of Ar (100 sccm) and CH_4_ (10–50 sccm). The CH_4_ gas (i.e., carbon source) was flown into the furnace at only the maximum annealing temperature. The furnace was then turned off, and the sample was also naturally cooled down in an Ar gas atmosphere. Finally, the sample was taken out of the furnace when the temperature of the furnace was below 100 °C.

### Characterizations

The morphologies of the carbon allotropes were characterized by a field emission scanning electron microscope (FESEM, JSM-7610F, Jeol) equipped with an energy-dispersive X-ray spectroscopy (EDX, X-Max^N^, Oxford Instruments). The size distribution, lattice spacing, and height of the synthesized carbon allotropes were also analyzed by a high-resolution transmission electron microscope (HRTEM, JEM-2100F, Jeol, 200 kV) with the Gatan software and an atomic force microscope (AFM, XE70, Park systems), respectively. The fast Fourier transform (FFT) image was obtained by processing using the Digital Micrograph (Gatan) software. The crystallinity of the carbon allotropes was measured by a Raman spectroscopy using a high-resolution spectrometer (i.e., ARAMIS, Horiba Jobin Yvon) with an excitation wavelength of 514.5 nm (i.e., Ar ion laser). The edge functional groups and chemical composition of the carbon allotropes were characterized from the X-ray photoelectron spectroscopy (XPS) measurements using an Al Kα source (K-alpha, Thermo VG Scientific).

## Results and discussion

**Fig. 1:**
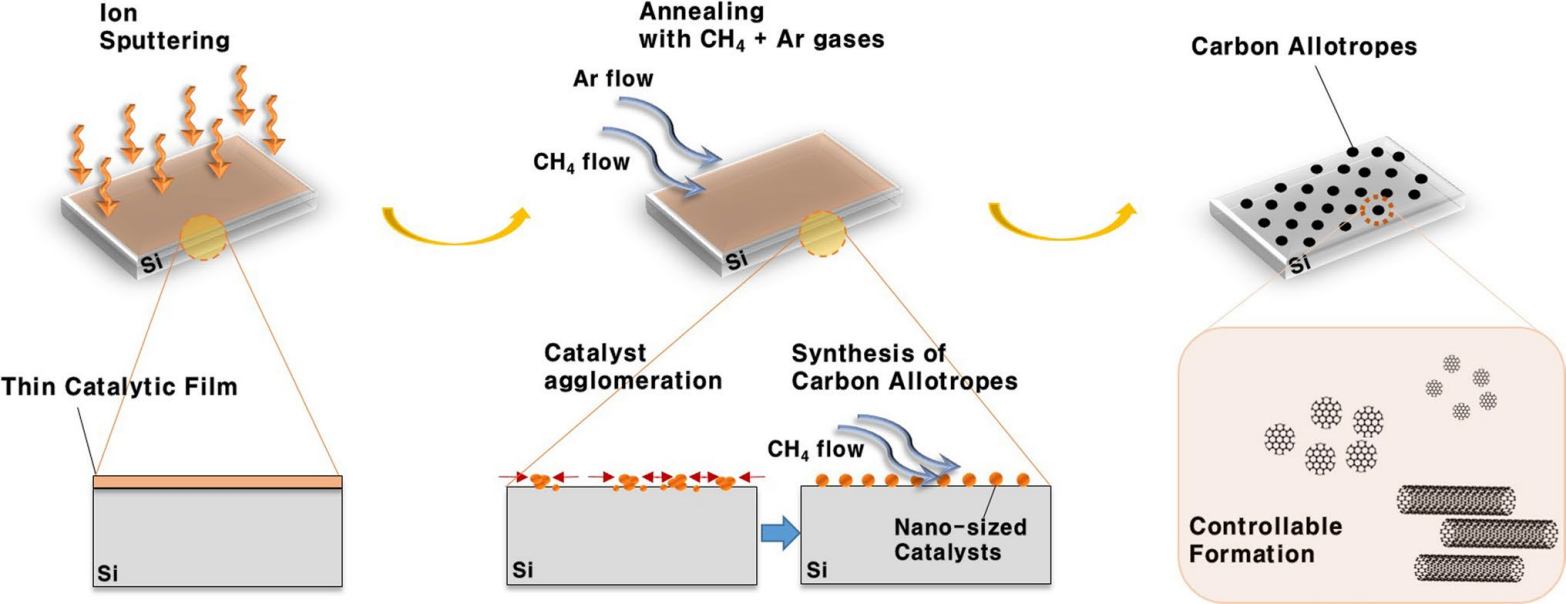
A schematic layout of a synthetic process for the carbon allotropes through our ion-sputtering assisted CVD method. During the annealing process, the deposited Pt thin film was changed to PtNPs, which acted as catalysts to synthesize from GQDs to CNTs

The carbon allotropes were fabricated by an ion-sputtering assisted CVD method that combined both the advantages of the ion-sputtering and CVD techniques, as schematically shown in Fig. 1. When the PtDS was annealed at a sufficiently high temperature, the deposited Pt thin film was dewetted and agglomerated to form many nanometer-sized particles. This led to the formation of PtNPs, which can act as catalysts to grow carbon allotropes. The shape of the allotropes can be effectively tailored from GQDs to CNTs by controlling the formation condition.

**Fig. 2:**
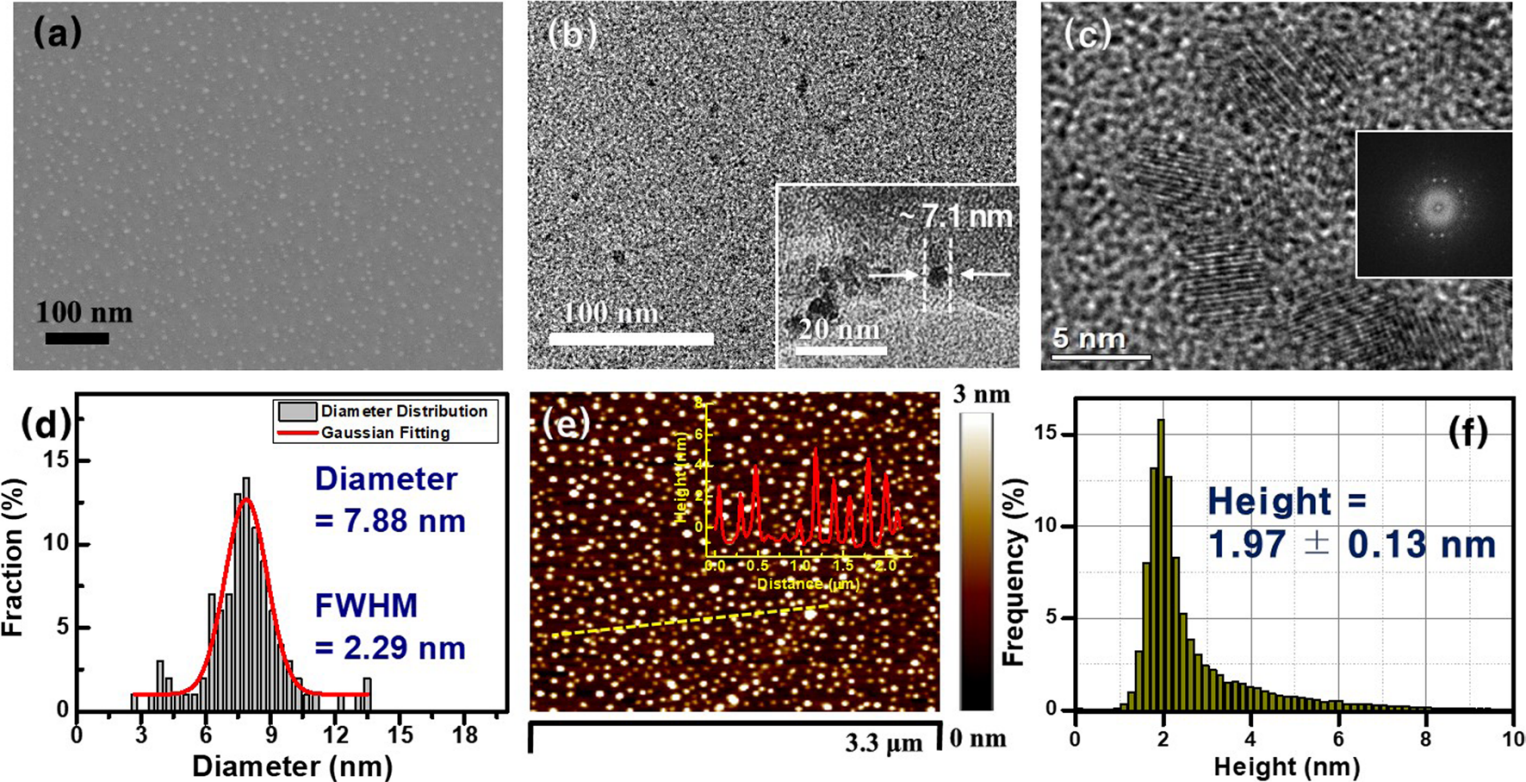
**a** FESEM and **b**, **c** TEM images of the GQDs. The size of the fabricated GQDs is ~ 7.1 nm (inset of **b**). **c** The magnified TEM image of the GQDs and its corresponding FFT pattern (inset). **d** The diameter distribution of the GQDs. The red line is the Gaussian fit curve. The average size of the GQDs and the full width at half-maximum (FWHM) of the Gaussian curve are 7.88 and 2.29 nm, respectively. **e** A typical AFM image of the GQDs. The height distribution of the GQDs in **e** and the measured location is indicated by a yellow-dashed line (inset). **f** Height distribution of the fabricated GQDs on a whole surface of the Si substrate

The FESEM, TEM, and AFM data display the GQDs prepared by our ion-sputtering assisted CVD method shown in Fig. 2. Pt ions were deposited on Si substrates through the ion-sputtering with an energy of 2 keV and a dose of 6.2 × 10^13^ #/cm^2^. When the PtDS was annealed at 900 °C for 20 min with a gas mixture of Ar (100 sccm) and CH_4_ (25 sccm), small-sized GQDs newly appeared on the surface of the Si substrate (Fig. 2a). The TEM and magnified images (Fig. 2b, c) obviously showed fabricated GQDs with a small spot shape. Furthermore, the HRTEM image and FFT pattern of the GQDs (Fig. 2c and inset) reveal that they had a crystalline structure [4, 21, 28] and a uniform particle size with an average diameter of 7.88 ± 2.29 nm (Fig. 2d). Figure 2e shows the typical AFM image of the GQDs, and the measured location is indicated by the yellow-dashed line representing randomly chosen GQDs. From the AFM data of the GQDs shown in Fig. 2e, f, it can be seen that the heights of the GQDs ranged from 0.5 to 4.4 nm (inset of Fig. 2e), and the average value was 1.97 ± 0.13 nm (Fig. 2f). A HRTEM image of the GQDs displayed lattice fringes with ~ 0.33 nm spacing ( Fig. S1), which corresponded to (002) planes of graphite [10, 14, 24], suggesting that the GQDs have a disk-shaped morphology and consist of a few layers of graphene [4, 24, 40, 41].

**Fig. 3:**
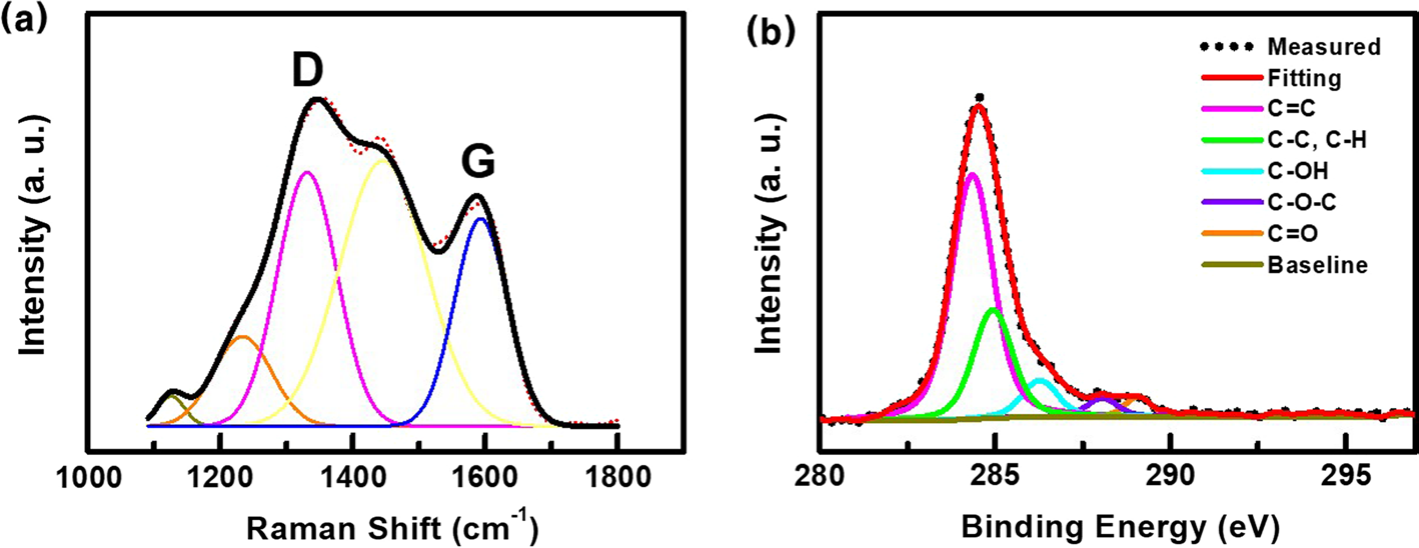
**a** Raman spectrum of the GQDs fitted with Gaussian peaks. **b** The C 1*s* XPS spectrum of the GQDs measured on a Si substrate. The peak consists of one main component arising from the C=C (284.33 eV) group and four minor components from the C–C, C–H (284.93 eV), C–OH (286.28 eV), C–O–C (288.03 eV), and C=O (289.08 eV) groups, respectively

Figure 3 exhibits the structural characterization of GQDs prepared by our ion-sputtering assisted CVD method. The Raman spectrum of the GQDs displays two main peaks at 1331 and 1591 cm^−1^ (Fig. 3a), respectively, which correspond to the D and G peaks of graphene [24, 25, 28]. The Raman intensity ratio of I_D_/I_G_ was ~ 1.4, which was attributed to the presence of a relatively high ratio of defects and edges in the GQDs as a result of their small lateral size [4, 12, 28]. Three weak peaks were observed at 1124, 1234, and 1445 cm^−1^, indicating that our GQDs included some proportions of glassy carbon structures in them [42, 43]. XPS analysis was performed to investigate the chemical structure of the GQDs. The C1s peak in the XPS spectra of the GQDs (Fig. 3b) shows that a small amount of C–C, C–H, C–OH, C–O–C, and C=O bonds exist within the main sp2 carbon (C=C) structure, which is the elementary unit of graphene.

**Fig. 4:**
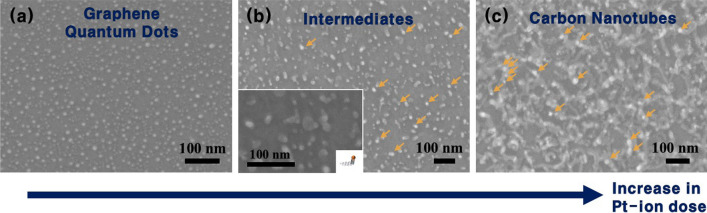
FESEM images of the carbon allotropes synthesized by different amounts of Pt ions with doses of **a** 7.4 × 10^13^, **b** 8.6 × 10^13^, and 9.8 × 10^13^ #/cm^2^, respectively. The Pt remnants (bright white-spots) are indicated by orange arrows in **b**, **c**. Insets: schematics and magnified FESEM images

The shape of the carbon allotropes can be effectively controlled by changing the dose of Pt ions and the annealing conditions. To investigate the effects of a Pt-ion dose on the growth of carbon allotropes, the allotropes were fabricated using different amounts of Pt ions. Figure 4 displays the FESEM images of the carbon allotropes synthesized at 900 °C for 20 min with a gas mixture of Ar (100 sccm) and CH_4_ (25 sccm). The doses of the Pt ions deposited on the Si substrates were 7.4 × 10^13^, 8.6 × 10^13^, and 9.8 × 10^13^ #/cm^2^, as shown in Fig. 4a–c, respectively. Compared to the small-sized GQDs (ion dose = 6.2 × 10^13^ #/cm^2^) in Fig. 2, as the dose of the Pt ions increased, the size of the resultant GQDs was larger, and finally CNTs were generated through an intermediate step (Fig. 4). It is interesting to note that there were numerous bump-like white-spots on the apexes of the CNTs (Fig. 4c), and a few white-spots also existed at the intermediate step (Fig. 4b). In addition,  Figure S2 shows the typical XPS survey spectra of the GQDs Fig. S2a and CNTs ( Fig. S2b), and the XPS measurements were performed more than 20 times for the randomly chosen samples. The results of the XPS analysis clearly revealed that no Pt was left on the substrate, indicating the production of high-purity GQDs. On the other hand, the detectable Pt remained in the CNT samples.

**Fig. 5:**
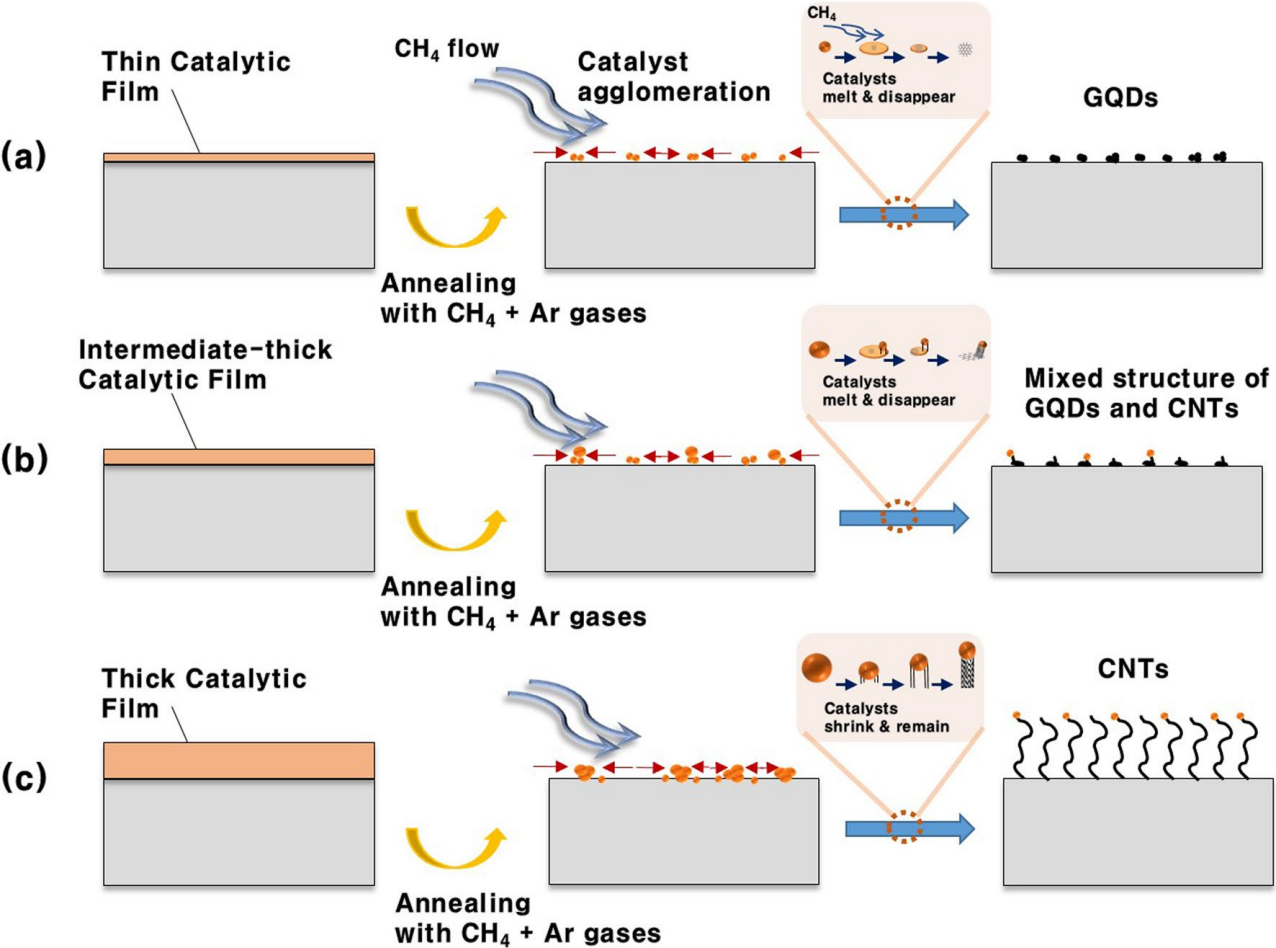
Formation mechanism of the **a** GQDs, **b** intermediates, and **c** CNTs in terms of the catalytic-ion dose

The formation mechanism of the carbon allotropes in terms of the catalytic-ion dose can be explained as follows. When the PtDS with a low dose of Pt ions (i.e., thin catalytic film on a substrate in Fig. 5a) was annealed at a sufficiently high temperature, the thin Pt film started to transform into small nanoparticles (i.e., < a few nanometers) and then the low cohesive energy of the small PtNPs causes separate Pt droplets to form on the substrate [28, 44–46]. Subsequently, after reacting with the carbon source, independent GQDs precipitate on the substrate, akin to the chemical vapor deposition (CVD) growth of graphene on liquid metal catalysts from hydrocarbon gas precursors [46–48]. Note that the Pt droplets are thermally evaporated and gradually removed from the substrate (Fig. 5a) [46]. This process results in the production of high-purity GQDs. When the PtDS with a high dose of Pt ions (i.e., thick catalytic film on a substrate in Fig. 5c) was annealed at the same condition, the relatively large PtNPs (> ~ 10 nm) induced by dewetting the thick Pt film were formed. Unlike the process for growing GQDs, the larger PtNPs were able to survive even at high temperatures in a vacuum due to their high cohesive energy [34, 37, 44, 45]. This enabled the catalytic graphitization process to be sustained at the annealing temperature, similar to the CVD growth of the CNTs using metal nanoclusters [33–35, 39]. As a result, the growth of CNTs with catalytic PtNP cap was achieved (Figs. 4c, 5c, and S4d) [49, 50]. These findings indicate that PtNPs serve as nucleation sites for the growth of carbon allotropes. Meanwhile, the mixed structure of GQDs and CNTs observed in Fig. 4b can be attributed to the intermediate amount of PtNPs used during synthesis. PtNPs in the intermediate step cannot be completely dissolved and a Pt particle remains due to their moderate size. As mentioned above, GQDs are generated from the molten Pt droplets, but CNTs grow from the Pt particle (Fig. 5b), which induced ultimately the mixture structure of GQDs and CNTs in the intermediate step.

Not only the dose of catalytic ions, but also the amount of carbon source and thermal energy were key parameters in controlling the shape of carbon allotropes. Figure S3 displays the carbon allotropes prepared by different amounts of the carbon source. Both the GQDs ( Fig. S3a) and CNTs ( Fig. S3b) were synthesized at the same condition, but only the amounts of the carbon source were different. When the allotropes were formed with a low amount of carbon source (CH_4_ 25 sccm), small spot-sized GQDs were grown on the substrate ( Fig. S3a). However, it can be distinctly seen that increasing the amount of carbon source produced elongated CNTs instead of GQDs, even under the same conditions (Fig. S3b). It clearly showed the effect of the amounts of carbon source on the shape control of the carbon allotropes. The structural characterizations of the CNTs measured by a FESEM, TEM, Raman spectroscopy, and XPS were described in the Supplementary Information (Fig. S4). In addition,  Figure S5 demonstrates that the thermal energy can also be a powerful parameter in tailoring the shape of the carbon allotropes. Increasing the annealing temperature accelerated the surface migration and thermal evaporation of the catalytic ions. It led to an early termination of the catalytic graphitization process [28, 33–35], resulting in smaller-sized GQDs ( Fig. S5a and S5b). Additionally, as can be inferred in the previous results shown in  Fig. S3, it was confirmed that the large-sized GQDs were generated ( Fig. S5c) when the amount of carbon source was increased under the same fabrication conditions with  Fig. S5b.

**Fig. 6:**
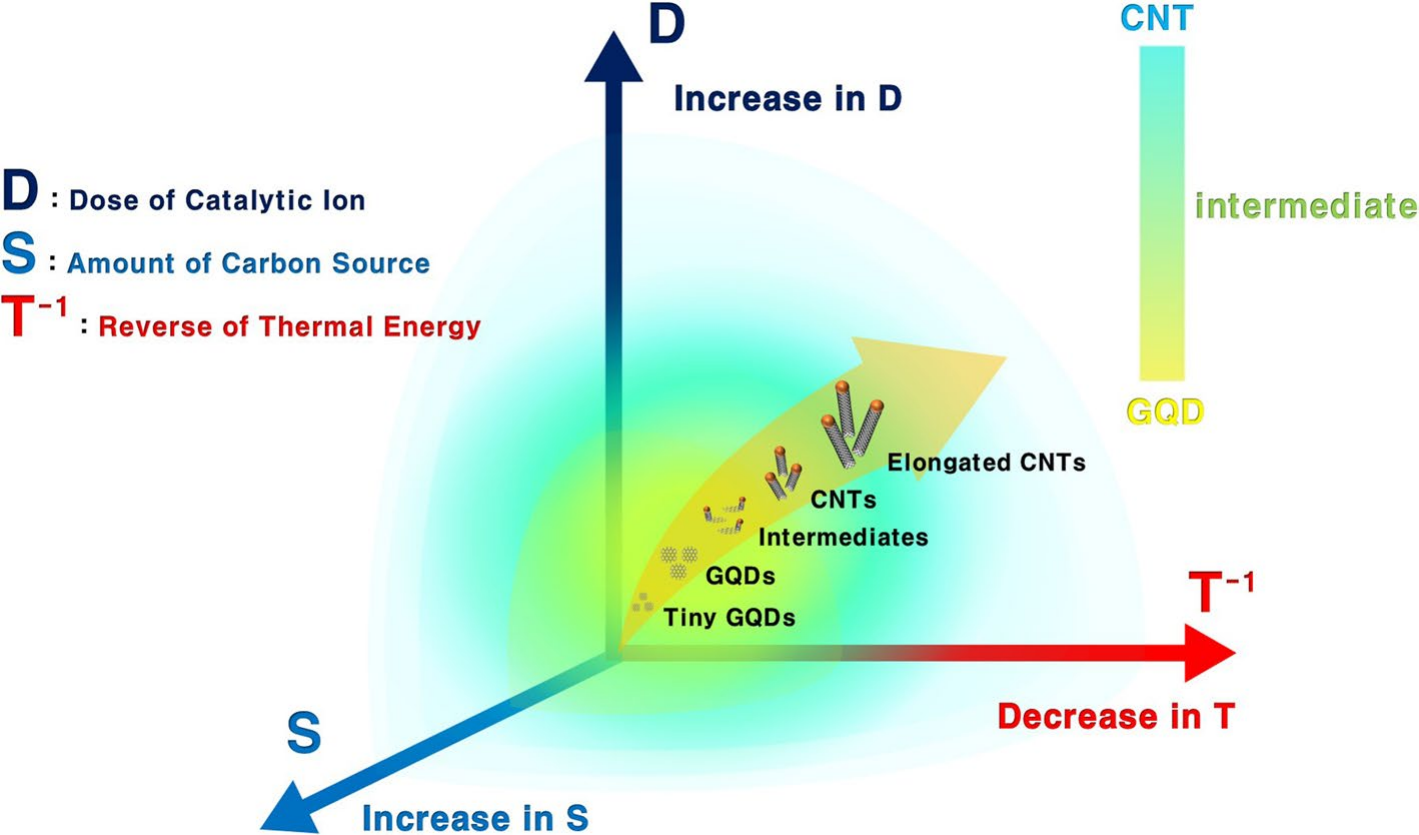
Key parameters for the controllable formation from GQDs to CNTs. The shape of carbon allotropes can be effectively tailored from GQDs to CNTs by controlling three key parameters such as the dose of catalytic ions (D), amounts of carbon source (S), and thermal energy (T)

The controllable formation from GQDs to CNTs for key parameters such as the dose of catalytic ions (D), amounts of carbon source (S), and thermal energy (T) can be clearly summarized as shown in Fig. 6. Based on the various data presented in this study, it can be inferred that the control of growth from GQDs to CNTs is comparatively proportional to D and S, but inversely proportional to T. In addition,  Figure S6 significantly proves our synthesis mechanism. As verified in  Fig. S5b and S5c, only GQDs were actually fabricated at 1050 °C, but when the D and S were simultaneously increased from the relationships inferred by Fig. 6, the CNTs were perfectly grown on the substrates instead of the GQDs (Fig. S6b and S6c).  Figure S6 reveals that adding variables that favor CNT synthesis can lead to preferential CNT growth, even under favorable conditions for the GQD growth. In conclusion, if our growth control technology, which consists of a combination of three key parameters, is well utilized, various carbon allotropes from GQDs to CNTs can be synthesized with the desired size, structure, and morphology. Moreover, intermediate region may give rise to a scientific concern liked to the quantum criticality of the metal–insulator transition, as GQDs exhibit insulating properties while CNTs demonstrate metallic behavior [36, 37].

## Conclusions

We have presented a simple and convenient route to the synthesis mechanism from GQDs to CNTs by using an ion-sputtering assisted one-step annealing process. During the thermal annealing, the deposited Pt thin film changed to PtNPs, which act as catalysts and nucleation sites to synthesize carbon allotropes. Our synthesis method enables the controlled creation of carbon allotropes with the desired size, structure, and morphology by manipulating three key parameters (D, S, and T). The validity of these parameters was confirmed through experimental data conducted under various fabrication conditions. In addition, our bottom-up synthesis strategy makes it possible to generate pure GQDs and CNTs with the cap of PtNPs, which is highly desirable in applications to industrial- and bio-fields. Another thing to note here is that the present work is the first report of the synthesis mechanism from GQDs to CNTs. Therefore, we believe that the present approach is very useful for diverse applications such as optoelectronics, nanophotonics, and sensing, as well as energy or environmental-related applications.

### Supplementary Information

Additional file1 (DOCX 1152 kb)

## Data Availability

The datasets generated during and/or analyzed during the current study are available from the corresponding author on reasonable request.

## References

[1] Hirsch A (2010). The era of carbon allotropes. Nat Mater.

[2] Liu Z, Ji X, He D, Zhang R, Liu Q, Xin T (2022). Nanoscale drug delivery systems in glioblastoma. Nanoscale Res Lett.

[3] Ye X, Qi M, Chen M, Zhang L, Zhang J (2023). Zero to three dimension structure evolution from carbon allotropes to phosphorus allotropes. Adv Mater Interfaces.

[4] Ye R, Xiang C, Lin J, Peng Z, Huang K, Yan Z, Cook NP, Samuel ELG, Hwang C, Ruan G, Ceriotti G, Raji AO, Martí AA, Tour JM (2013). Coal as an abundant source of graphene quantum dots. Nat Commun.

[5] Semeniuk M, Yi Z, Poursorkhabi V, Tjong J, Jaffer S, Lu Z, Sain M (2019). Future perspectives and review on organic carbon dots in electronic applications. ACS Nano.

[6] Chung S, Revia RA, Zhang M (2021). Graphene quantum dots and their applications in bioimaging, biosensing, and therapy. Adv Mater.

[7] Ha JM, Kim HJ, Raza HS, Cho SO (2013). Highly stable carbon nanotube field emitters on small metal tips against electrical arcing. Nanoscale Res Lett.

[8] Rathinavel S, Priyadharshini K, Panda D (2021). A review on carbon nanotube: an overview of synthesis, properties, functionalization, characterization, and the application. Mater Sci Eng B.

[9] Sun D, Liu C, Ren W, Cheng H (2013). A review of carbon nanotube- and graphene-based flexible thin-film transistors. Small.

[10] Luk CM, Tang LB, Zhang WF, Yu SF, Teng KS, Lau SP (2012). An efficient and stable fluorescent graphene quantum dot-agar composite as a converting material in white light emitting diodes. J Mater Chem.

[11] Zhu S, Zhang J, Qiao C, Tang S, Li Y, Yuan W, Li B, Tian L, Liu F, Hu R, Gao H, Wei H, Zhang H, Sun H, Yang B (2011). Strongly green-photoluminescent graphene quantum dots for bioimaging applications. Chem Commun.

[12] Carrasco PM, García I, Yate L, Zaera RT, Cabanero G, Grande HJ, Ruiz V (2016). Graphene quantum dot membranes as fluorescent sensing platforms for Cr (VI) detection. Carbon.

[13] Chen H, Wang Z, Zong S, Chen P, Zhu D, Wu L, Cui Y (2015). A graphene quantum dot-based FRET system for nuclear-targeted and real-time monitoring of drug delivery. Nanoscale.

[14] Li H, He X, Kang Z, Huang H, Liu Y, Liu J, Lian S, Tsang CHA, Yang X, Lee S (2010). Water-soluble fluorescent carbon quantum dots and photocatalyst design. Angew Chem Int Ed.

[15] Peng L, Zhang Z, Qiu C (2019). Carbon nanotube digital electronics. Nat Electron.

[16] Alosime EM (2023). A review on surface functionalization of carbon nanotubes: methods and applications. Discover Nano.

[17] Peretz S, Regev O (2012). Carbon nanotubes as nanocarriers in medicine. Curr Opin Colloid Interface Sci.

[18] De Volder MFL, Tawfick SH, Baughman RH, Hart AJ (2013). Carbon nanotubes: present and future commercial applications. Science.

[19] Markovic ZM, Ristic BZ, Arsikin KM, Klisic DG, Trajkovic LMH, Markovic BMT, Kepic DP, Stevovic TKK, Jovanovic SP, Milenkovic MM, Milivojevic DD, Bumbasirevic VZ, Dramicanin MD, Trajkovic VS (2012). Graphene quantum dots as autophagy-inducing photodynamic agents. Biomaterials.

[20] Xu X, Ray R, Gu Y, Ploehn HJ, Gearheart L, Raker K, Scrivens WA (2004). Electrophoretic analysis and purification of fluorescent single-walled carbon nanotube fragments. J Am Chem Soc.

[21] Moon J, An J, Sim U, Cho SP, Kang JH, Chung C, Seo JH, Lee J, Nam KT, Hong BH (2014). One-step synthesis of N-doped graphene quantum sheets from monolayer graphene by nitrogen plasma. Adv Mater.

[22] Ciesielski A, Haar S, Aliprandi A, Garah ME, Tregnago G, Cotella GF, Gemayel ME, Richard F, Sun H, Cacialli F, Bonaccorso F, Samorì P (2016). Modifying the size of ultrasound-induced liquid-phase exfoliated graphene: from nanosheets to nanodots. ACS Nano.

[23] Zhou J, Booker C, Li R, Zhou X, Sham TK, Sun X, Ding Z (2007). An electrochemical avenue to blue luminescent nanocrystals from multiwalled carbon nanotubes (MWCNTs). J Am Chem Soc.

[24] Luo Z, Qi G, Chen K, Zou M, Yuwen L, Zhang X, Huang W, Wang L (2016). Microwave-assisted preparation of white fluorescent graphene quantum dots as a novel phosphor for enhanced white-light-emitting diodes. Adv Funct Mater.

[25] Yu R, Liang S, Ru Y, Li L, Wang Z, Chen J, Chen L (2022). A facile preparation of multicolor carbon dots. Nanoscale Res Lett.

[26] Shen CL, Su LX, Zang JH, Li XJ, Lou Q, Shan CX (2017). Carbon nanodots as dual-mode nanosensors for selective detection of hydrogen peroxide. Nanoscale Res Lett.

[27] Bourlinos AB, Stassinopoulos A, Anglos D, Zboril R, Georgakilas V, Giannelis EP (2008). Photoluminescent carbogenic dots. Chem Mater.

[28] Ha JM, Lee NE, Yoon YJ, Lee SH, Hwang YS, Suk JK, Lee CY, Kim CR, Yeo S (2022). Universal dry synthesis and patterning of high-quality and -purity graphene quantum dots by ion-beam assisted chemical vapor deposition. Carbon.

[29] Liu F, Chen X, Liu H, Zhao J, Xi M, Xiao H, Lu T, Cao Y, Li Y, Peng L, Liang X (2021). High-yield and low-cost separation of high-purity semiconducting single-walled carbon nanotubes with closed-loop recycling of raw materials and solvents. Nano Res.

[30] Zhang Y, Gu H, Iijima S (1998). Single-wall carbon nanotubes synthesized by laser ablation in a nitrogen atmosphere. Appl Phys Lett.

[31] Das R, Shahnavaz Z, Ali ME, Islam MM, Hamid SBA (2015). Can we optimize arc discharge and laser ablation for well-controlled carbon nanotube synthesis?. Nanoscale Res Lett.

[32] Alamro FS, Mostafa AM, Al-Ola KAA, Ahmed HA, Toghan A (2021). Synthesis of Ag nanoparticles-decorated CNTs via laser ablation method for the enhancement the photocatalytic removal of naphthalene from water. Nanomaterials.

[33] Kim SM, Pint CL, Amama PB, Zakharov DN, Hauge RH, Maruyama B, Stach EA (2010). Evolution in catalyst morphology leads to carbon nanotube growth termination. J Phys Chem Lett.

[34] Li H, Yuan G, Shan B, Zhang X, Ma H, Tian Y, Lu H, Liu J (2019). Chemical vapor deposition of vertically aligned carbon nanotube arrays: critical effects of oxide buffer layers. Nanoscale Res Lett.

[35] Stadermann M, Sherlock SP, In J, Fornasiero F, Park HG, Artyukhin AB, Wang Y, Yoreo JJD, Grigoropoulos CP, Bakajin O, Chernov AA, Noy A (2009). Mechanism and kinetics of growth termination in controlled chemical vapor deposition growth of multiwall carbon nanotube arrays. Nano Lett.

[36] Li Z, Jiang Y, Jian S, Yao H (2017). Fermion-induced quantum critical points. Nat Commun.

[37] Son DT (2007). Quantum critical point in graphene approached in the limit of infinitely strong Coulomb interaction. Phys Rev B.

[38] Sui M, Li M, Kunwar S, Pandey P, Zhang Q, Lee J (2017). Effects of annealing temperature and duration on the morphological and optical evolution of self-assembled Pt nanostructures on c-plane sapphire. PLoS ONE.

[39] Lee C, Lee J, Yeo S, Lee S, Kim T, Cha H, Eun Y, Park HJ, Kim SM, Lee K (2017). Evolution of implanted Fe ions in SiO_2_/Si wafer into uniformly sized catalyst particles for carbon nanotube forest growth. Carbon.

[40] Tang L, Ji R, Cao X, Lin J, Jiang H, Li X, Teng KS, Luk CM, Zeng S, Hao J, Lau SP (2012). Deep ultraviolet photoluminescence of water-soluble self-passivated graphene quantum dots. ACS Nano.

[41] Liu R, Wu D, Feng X, Müllen K (2011). Bottom-up fabrication of photoluminescent graphene quantum dots with uniform morphology. J Am Chem Soc.

[42] Jurkiewicz K, Pawlyta M, Zygadło D, Chrobak D, Duber S, Wrzalik R, Ratuszna A, Burian A (2018). Evolution of glassy carbon under heat treatment: correlation structure-mechanical properties. J Mater Sci.

[43] Hong S, Winter J (2005). Micro-Raman spectroscopy on a-C: H nanoparticles. J Appl Phys.

[44] Guisbiers G, Abudukelimu G, Hourlier D (2011). Size-dependent catalytic and melting properties of platinum-palladium nanoparticles. Nanoscale Res Lett.

[45] Wang G, Xu Y, Qian P, Su Y (2019). The effects of size and shape on the structural and thermal stability of platinum nanoparticles. Comput Mater Sci.

[46] Ha JM, Lim HS, Park JW, Kim HJ, Cho SO (2016). Freestanding graphene nanosheets and large-area/patterned graphene nanofilms from indiumcatalyzed graphite. RSC Adv.

[47] Liu J, Fu L (2019). Controllable growth of graphene on liquid surfaces. Adv Mater.

[48] Ding G, Zhu Y, Wang S, Gong Q, Sun L, Wu T, Xie X, Jiang M (2013). Chemical vapor deposition of graphene on liquid metal catalysts. Carbon.

[49] Han JH, Choi SH, Lee TY, Yoo JB, Park CY, Jung T, Yu SG, Yi W, Han IT, Kim JM (2003). Growth characteristics of carbon nanotubes using platinum catalyst by plasma enhanced chemical vapor deposition. Diam Relat Mater.

[50] Brown B, Parker CB, Stoner BR, Glass JT (2011). Growth of vertically aligned bamboo-like carbon nanotubes from ammonia/methane precursors using a platinum catalyst. Carbon.

