# Supplementary material for: Synthesis mechanism from graphene quantum dots to carbon nanotubes by ion-sputtering assisted chemical vapor deposition
Source: Discov Nano. 2024 May 7;19(1):83. doi: 10.1186/s11671-024-04027-3 (PMC11076437; doi:10.1186/s11671-024-04027-3)
Supplement: Supplementary file 1 — Additional file1 (DOCX 1152 kb) [file 11671_2024_4027_MOESM1_ESM.docx]

Supplementary Information

**Synthesis mechanism from graphene quantum dots to carbon nanotubes by ion-sputtering assisted chemical vapor deposition**

Jun Mok Ha ^a,^^[[1]](#footnote-1)^1, Seoung Ho Lee ^b,1^, Daehyeon Park ^c^, Young Jun Yoon ^a^, In Mok Yang ^a^, Junhyeok Seo ^a,d^, Yong Seok Hwang ^a^, Chan Young Lee ^a^, Jae Kwon Suk ^a^, Jun Kue Park ^a^, and Sunmog Yeo ^a,^^[[2]](#footnote-2)^

^a^ Particle Beam Research Division, Korea Multi-purpose Accelerator Complex (KOMAC), Korea Atomic Energy Research Institute (KAERI), 181 Mirae-ro, Geonchon-eup, Gyeongju-si, Gyeonbuk 38180, Republic of Korea

^b^ Department of Material & Equipment Development, Korea Research Institute of Decommissioning (KRID), 1655 Bulguk-ro, Munmudaewang-myeon, Gyeongju-si, Gyeongbuk 38120, Republic of Korea

^c^ Department of Nuclear Engineering, College of Engineering, Ulsan National Institute of Science and Technology (UNIST), 50 UNIST-gil, Ulsan 44919, Republic of Korea

^d^ Department of Electrical and Electronic Engineering, Hannam University, 70 Hannam-ro, Daedeok-gu, Daejeon 34430, Republic of Korea

**Keywords:** Graphene Quantum Dots, Carbon Nanotubes, Controllable Formation, Ion-sputtering, Chemical Vapor Deposition, Platinum Nanoparticles

**HRTEM analysis of GQDs**

**
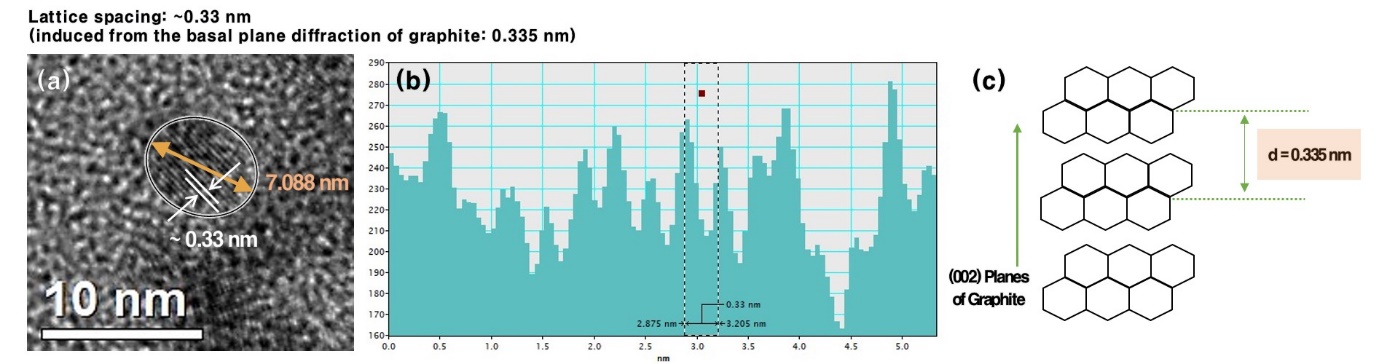
**

**Fig. S1.** HRTEM images and line profile analysis of the GQDs. (a) The HRTEM image of the GQD and (b) its corresponding line profile analysis (indicated by orange line). The size and lattice spacing of GQDs are 7.088 and ~0.33 nm, respectively. (c) The lattice fringe (d = 0.335 nm) is induced from the basal plane diffraction of graphite.

**XPS analysis of GQDs and CNTs**

**
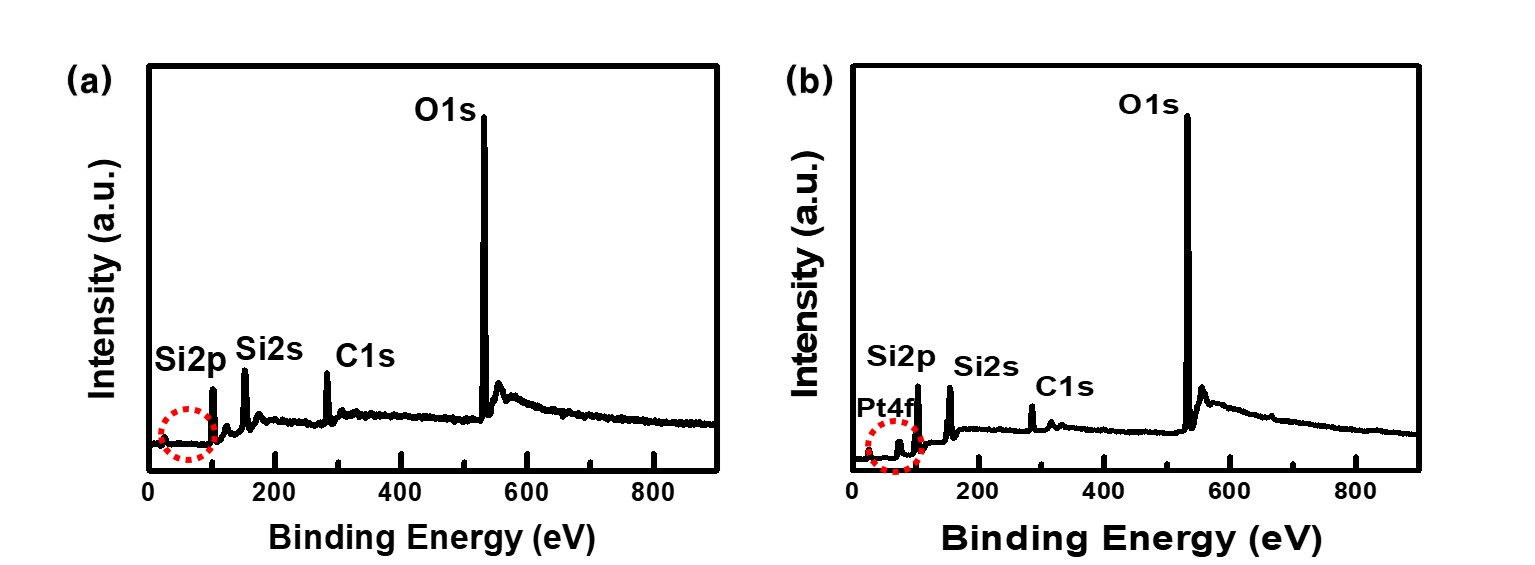
**

**Fig. S2.** Typical XPS spectra of the (a) GQDs and (b) CNTs on Si substrates, respectively. The positions of Pt4f peak are indicated by red circles in (a) and (b). The XPS measurements were performed more than 20 times for the randomly chosen samples.

**FESEM images of the carbon allotropes synthesized by different amounts of the carbon source**


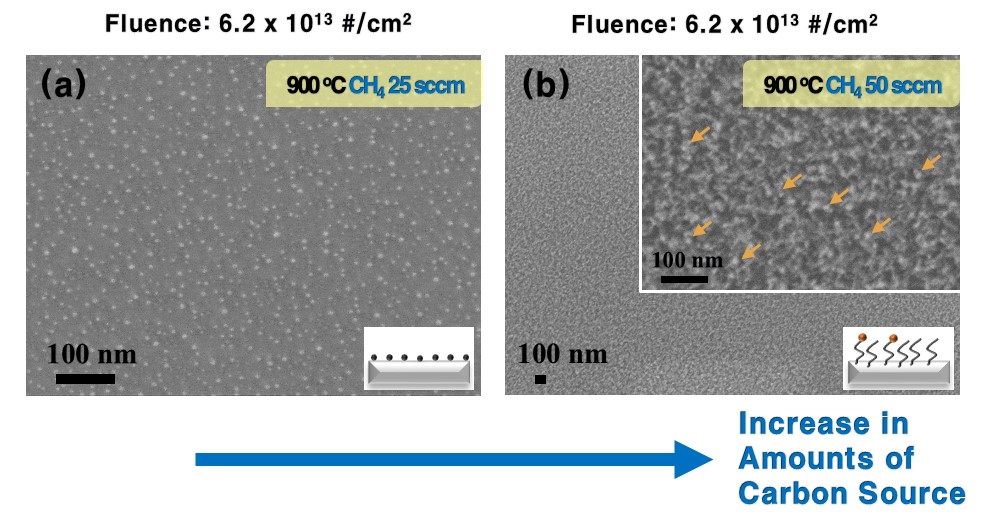


**Fig. S3.** FESEM images of the carbon allotropes synthesized by different amounts of the carbon source with (a) CH_4_ 25 sccm and (b) 50 sccm, respectively. Insets: schematics and a magnified FESEM image of the fabricated carbon allotropes. The Pt remnants (bright white-spots) are indicated by orange arrows in the inset of (b).

**Structural characterizations of the CNTs**

**
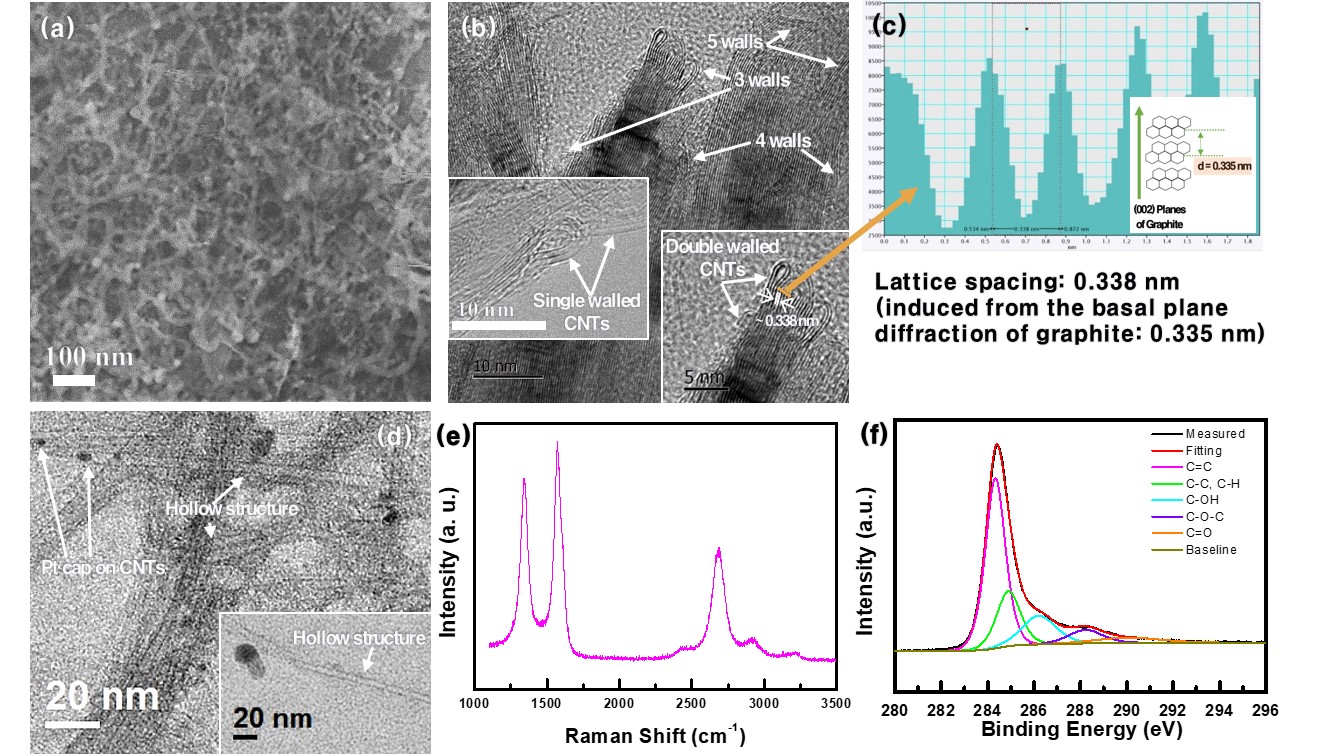
**

**Fig. S4.** Structural characterization of the CNTs. (a) The FESEM and (b,d) TEM images of the CNTs and (c) its corresponding line profile analysis (indicated by orange line in the inset of (b). Insets: magnified TEM images of the fabricated CNTs. The CNTs (with their walls) and Pt caps are indicated by arrows in (b) and (d), respectively. (e) Raman and (f) C1s XPS spectra of the CNTs.

Pt ions were deposited on Si substrates through the ion-sputtering with an energy of 2 keV and a dose of 5 × 10^13^ ~ 1× 10^14^ #/cm^2^. When the PtDS was annealed at 900 ^o^C for 20 min with a gas mixture of Ar (100 sccm) and CH_4_ (50 sccm), strip-shaped CNTs newly synthesized (Fig. S4). Fig. 4a-d display the FESEM and TEM images of fabricated CNTs. The CNTs had 1-7 walls on average, and mainly composed of few-walled or multi-walled CNTs with 3-5 walls (Fig. S4b). The HRTEM image of the CNTs shown in Fig. S4b and c reveals that they have a highly-ordered crystalline structure and lattice fringes with ~0.338 nm spacing, which corresponds to the basal-plane distance of graphite (Fig. S4c). Furthermore, Fig. S4d shows that the CNTs have a hollow structure and some CNTs exist with Pt caps on the tips. However, there are some CNTs with missing Pt caps due to the high temperature process. The Raman spectrum of the CNTs displays three main peaks at 1343, 1572, and 2688 cm^-1^ (Fig. S4e), which correspond to the D, G, and 2D peaks of typical multi-layer CNTs. The C1s peak in the XPS spectra of the CNTs (Fig. S4f) exhibits that a small amount of C-C, C-H, C-OH, C-O-C, and C=O bonds exist within the main sp2 carbon (C=C) structure.

**FESEM images of the carbon allotropes synthesized at different temperatures**


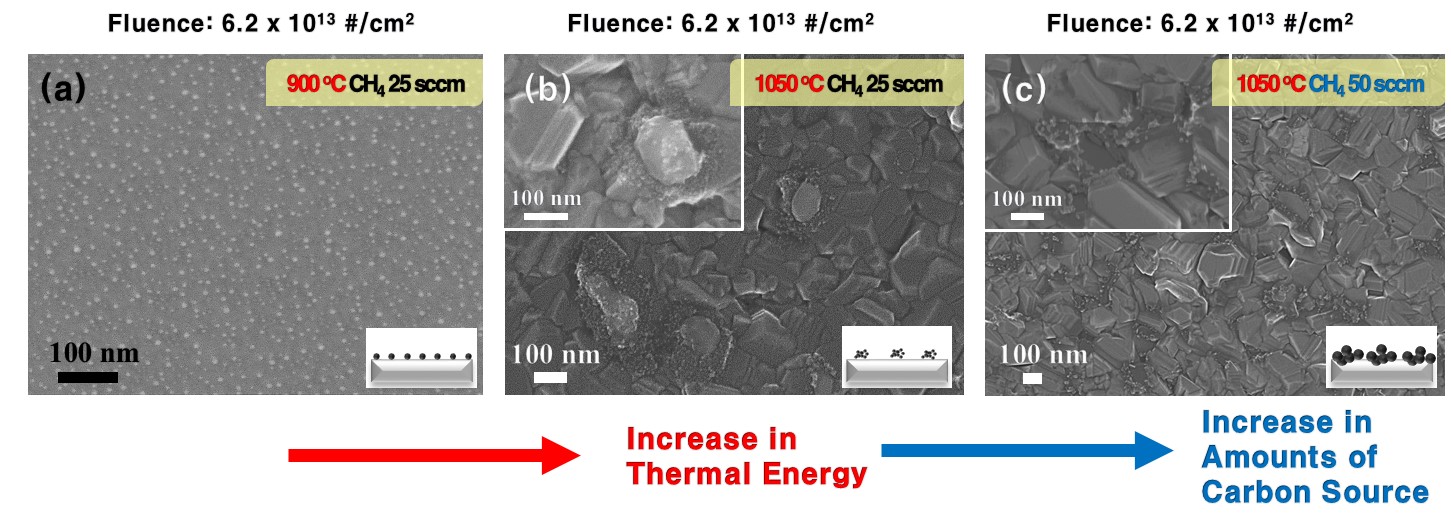


**Fig. S5.** FESEM images of the carbon allotropes synthesized at different temperatures of (a) 900 ^o^C and ((b) and (c)) 1050 ^o^C, respectively. (c) The FESEM image of the GQDs grown with an increased carbon source of CH_4_ 50 sccm. Insets: schematics and magnified FESEM images of the fabricated carbon allotropes.

**FESEM images of the carbon allotropes synthesized at different fabrication conditions**


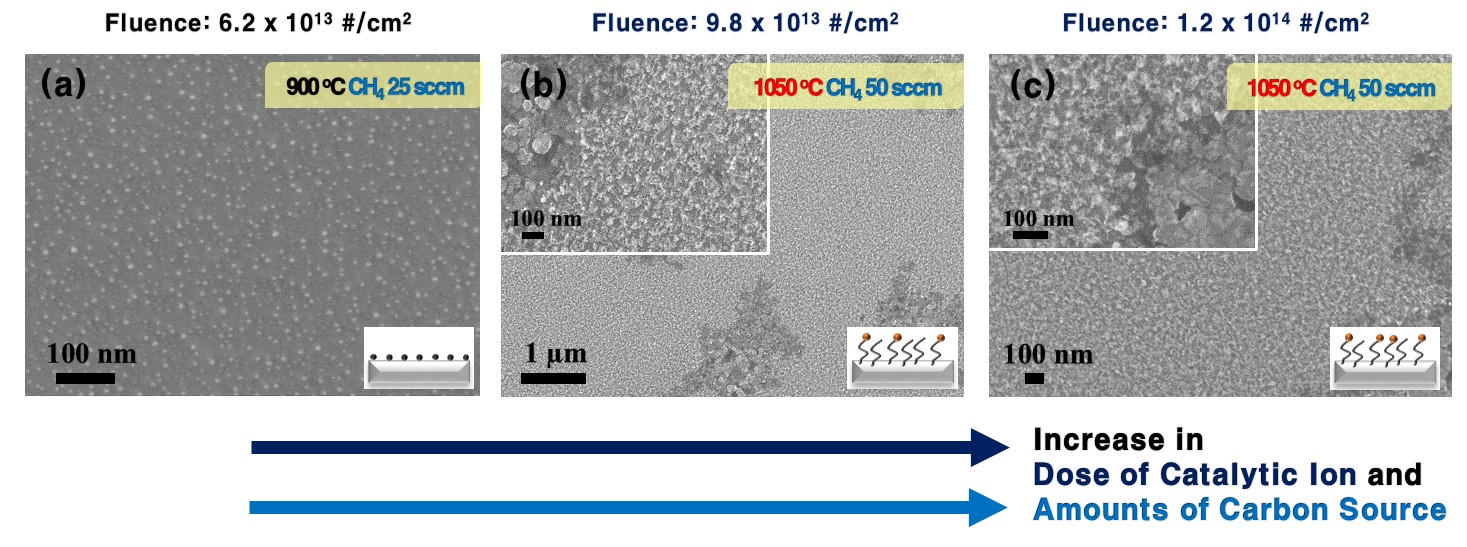


**Fig. S6.** FESEM images of the carbon allotropes synthesized at different fabrication conditions. (a) The FESEM image and schematic (inset) of the GQDs grown with low dose of catalytic ion (6.2 x 10^13^ #/cm^2^) and amounts of carbon source (CH_4_ 25 sccm) at an annealing temperature of 900 ^o^C. ((b) and (c)) FESEM, magnified FESEM images (insets) and schematics (insets) of the CNTs fabricated at 1050 ^o^C with same amounts of carbon source (CH_4_ 50 sccm) but different Pt-ion doses of (b) 9.8 x 10^13^ #/cm^2^ and (c) 1.2 x 10^14^ #/cm^2^, respectively.

1. 1 Co-first Authors. These authors contributed equally to this work. [↑](#footnote-ref-1)
2. Corresponding Author. Tel/Fax: +82-54-750-5309/+82-54-750-5715. Email address: [sunmog@kaeri.re.kr](mailto:sunmog@kaeri.re.kr) (**Sunmog Yeo**) [↑](#footnote-ref-2)
